# Extending the Dark Side of Identity Processes With Identity Distress

**DOI:** 10.1002/jad.70034

**Published:** 2025-08-11

**Authors:** Barbara M. Gfellner, Karin Bartoszuk, Jim Deal

**Affiliations:** ^1^ Brandon University Brandon Manitoba Canada; ^2^ East Tennessee State University Johnson City Tennessee USA; ^3^ North Dakota State University Fargo North Dakota USA

**Keywords:** Canadian students, DIDS, ego strengths, identity distress, latent profile analysis, psychological symptoms

## Abstract

**Introduction:**

Latent profile analysis of the dimensions of identity scale (DIDS) provides a person‐centered configuration of adaptive (commitment, identification with commitment; exploration in breadth and in depth) and maladaptive (ruminative exploration) identity processes. The DIDS focuses on the future domain in the present. In this study DIDS profile analysis was extended to include the Identity Distress Scale (IDS), an affective index of uncertainty and difficulties with identity issues in general content areas relevant to young adults and its impact on adjustment. Identity distress is conceptualized on a continuum from adaptive to dysfunctional in relation to identity development. Its inclusion with identity processes was expected to provide a more nuanced description of the person‐centered identity profiles.

**Methods:**

Participants were 914 students (mdn = 19 years; 79.6% women) at a university located in a nonmetropolitan city in the prairie provinces of Canada. During 2017 and 2018 students completed an online survey that included the DIDS, IDS, and various standardized measures of identity, mental health, and adjustment at university.

**Results:**

The findings revealed four latent profiles: Carefree Diffusion (6.9%), Troubled Diffusion (24.8%), Achievement (28.2%), and Undifferentiated (40.1%) but neither Foreclosure nor Moratorium were found. These results are consistent with variations in the profiles across other countries and contexts. Expected associations were supported between the profiles and most outcome measures. The low incidence of adaptive identity processes notably exploration was considered in terms of culture and context.

**Conclusions:**

This study adds to the complexity of individual pathways and contextual differences in identity development.

## 1 Introduction

Identity development is the primary psychosocial task of adolescents and young adults (Erikson 1968). It involves exploring and evaluating life choices in the process of constructing a coherent and integrated sense of self. It is the turning point that includes consolidating a conceptualization of *who I am*, with *where I have been*, and *where I am going*. Identity theory has evolved with conceptual refinements in conjunction with contextual changes in contemporary societies (Schwartz 2005, 2016; Schwartz et al. 2013) and has remained the cornerstone of well‐being (Crocetti et al. 2016; Kroger and Marcia 2022; Kroger and Marcia 2011; Luyckx et al. 2011). This study used latent profile analysis of the dimensions of identity scales (DIDS; Luyckx and colleagues 2006; 2008a; Schwartz et al. 2011) with the identity distress scale (IDS; Berman et al. 2004) to expand the affective component of the paradigm.

Identity processes on the DIDS index commitment, adaptive and maladaptive exploration with a focus on the future in the present without reference to content. Alternatively, identity distress on the IDS assesses the extent to which individuals have recently experienced upset, distress, discomfort, or worry in several identity domains and the overall impact of this uncertainty on daily functioning. Identity distress reflects the affective aspect of maladaptive exploration that falls along a continuum from normative to dysfunctional (Berman and Montgomery 2014; Klimstra and Denissen 2017). In this study the IDS in conjunction with the DIDS adds the valence and impact of identity content to the person‐centered configurations to provide a more comprehensive account of the identity profiles and associations with adjustment with implications for theory, research, and intervention.

## 2 Identity Development

Erikson's formulation of identity development was operationalized by Marcia (1966) in two dimensions: exploration (searching, evaluating, and questioning lifestyle alternatives) and commitment (selecting and adhering to specific choices). These were cross‐referenced into four identity statuses: diffusion (neither exploration nor commitment), foreclosure (commitment without exploration), moratorium (active involvement in exploration), and achievement (commitment after a period of exploration). The identity statuses have been studied extensively in terms of mental health and well‐being with comprehensive reviews available (cf. Kroger et al. 2010; Kroger and Marcia 2022, 2011).

Changes in modern societies led to refinement of these statuses to reflect increased complexities in the processes of identity development (e.g., Schwartz 2005; 2016; Luyckx et al. 2011). Luyckx and colleagues (2006; 2008a) unpacked Marcia's paradigm in the dual‐cycle model. The first cycle included Marcia's dimensions of exploration in breadth (examining potential options) and commitment making (adhering to choice). This was followed in the second cycle by exploration in depth (additional consideration and examining one's choices) and identification with commitment (reaffirming and reidentifying commitments). In addition to these adaptive processes, ruminative exploration was included to reflect dysfunctional exploration (Luyckx et al. 2008a). Ruminative exploration is characterized by brooding, lack of clarity, procrastination, and confusion that stifles appropriate exploration.

Recently person‐centered analysis has been used to determine more subtle complexities in identity processes in comparison with variable‐centered research (Crocetti and Meeus 2015). Studies using the DIDS (e.g., Luyckx et al. 2008a, 2008b; Schwartz et al. 2011) revealed additional configurations of identity processes (i.e., carefree diffusion, diffused diffusion, and undifferentiated). As with the identity statuses, positive adjustment is associated with profiles high in commitment and poor adjustment among those in profiles with elevated maladaptive exploration.

## 3 Identity Distress

In the modern world, the period of identity formation has been extended into the 20's and named “emerging adulthood” (EA; Arnett 2016). This age‐span reflects increased complexities associated with the increased time for education and delays in marriage and parenthood that defer role requirements associated with adulthood (Arnett 2016). EA provides more opportunities for young people to experiment with different options, yet there are fewer guidelines and direction so it is a time of instability and ambivalence about the future (Arnett 2016). A certain amount of distress and uncertainty with identity issues is normal. However, some individuals become overwhelmed to the extent that identity distress is dysfunctional and undermines everyday functioning (Berman et al. 2014, 2009).

From the clinical perspective, identity distress refers to severe difficulties, uncertainty, and confusion dealing with identity issues that interfere with adjustment (APA‐5 2013). Considerable research indicates its importance in the mental health and well‐being of community (Gfellner and Cordoba 2011; 2020a; Palmeroni et al. 2020) and clinical samples (Hernandez et al. 2006; Kamps and Berman 2011; Papazova et al. 2018; Samuolis et al. 2015; Scott et al. 2014; Wiley and Berman 2013). According to developmental psychopathology, identity distress falls along a continuum that extends from normal to severe disability (Berman and Montgomery 2014). It is the affective component in identity development associated with content relevant to EAs and maladaptive exploration (Gfellner and Cordoba 2017; Palmeroni et al. 2020).

## 4 Identity Development and Identity Distress

Identity distress is positively related to moratorium, the identity status that involves active exploration without commitment, and inversely associated with commitment (Berman and Montgomery 2014, 2014, 2009). In a study of Flemish adolescents and emerging adults, Palmeroni et al. (2020) reported negative correlations between identity distress with CM (−.16) and IC (−.24), a substantial positive correlation with RE (0.48), and lesser with EB (0.15) and ED (0.14). Consistent results were found among university students in Italy (Sica et al. 2014) and Canada (Gfellner and Cordoba 2020b).

Identity distress is an affective aspect of identity functioning that is directly and indirectly associated with RE and adjustment (Gfellner and Cordoba 2020b). It reflects respondents' uncertainty and difficulties with relevant content domains including future concerns. The inclusion of the IDS with the DIDS is expected to enhance the depiction of identity profiles.

## 5 Cultural Context of the Sample

According to Hofstede's (2015) cultural dimensions theory, Canada is an individualistic culture, more subdued with respect to achievement, success, and winning than the US, with emphasis on a work‐leisure/personal activities balance. Canada is an egalitarian, slightly pragmatic, and relatively progressive culture that adapts easily to changing conditions.

Canada is defined as a cultural mosaic that reflects pluralism in ethnic and cultural diversity. This is seen in regionalism ‐ the distinctive local characteristics of a geographical area or people's perception and identification with such places (The Canadian Encyclopedia 2021). In particular, the prairie provinces are less densely populated with smaller communities, more rural agriculture, and a greater proportion of people with eastern and central European backgrounds. Recent trends indicate fewer 18 to 24‐year‐olds living in their parents' home (56%) than the country overall (73%), even fewer in rural locations (Statistics Canada 2022), and the lowest age of marriage (Statistics Canada 2022).

## 6 The Current Study and Predictions

This study used latent profile analysis to investigate profiles of identity processes and identity distress in a sample of Canadian university students. As with previous research on person‐centered approaches with identity processes, relevant identity profiles were empirically constructed. (1) Following earlier studies (Luyckx et al. 2008a, 2008b; Schwartz et al. 2011), we anticipated replication of achievement, foreclosure, moratorium, troubled diffusion, carefree diffusion, and undifferentiated profiles. (2) Construct validity was examined in relation to standardized identity instruments. (3) The identity profiles were examined in relation to measures of functioning relevant to university students: mental health and adjustment at university. The predictions were that the mature identity profiles (high commitment) would be associated with elevated functioning on the identity measures, positive mental health, and functioning at university, respectively.

## 7 Methods

### 7.1 Participants

This study included 914 university students between 17 and 26 years of age (*M* = 19.4, SD = 2.1; 76.9% female). See Supporting Table 1 for a complete description.

### 7.2 Measures

The **Dimensions of Identity Development Scale** (DIDS; Luyckx et al. 2008b) was the measure of identity processes. The DIDS includes 25‐items with 5‐items for each of the five scales: commitment making (CM (*ω* = 0.90) e.g., “I know what I want to do with my life”); identification with commitments (IC (*ω* = 0.92) e.g., **“**My plans for the future offer me a sense of security”); exploration in breadth (EB (*ω* = 0.62) e.g., “I think a lot about the direction I want to take in my life”); exploration in depth (ED (*ω* = 0.72) e.g., “I think about the future plans I have made”); and ruminative exploration (RE (*ω* = 0.77) e.g., “I worry about what I want to do with my future”). Items are rated on a 5‐point scale (1 = “strongly disagree” to 5 = “strongly agree”) to indicate the extent to which they apply to the respondent. Psychometric properties are given in Luyckx et al. (2006; 2013) and others for different countries (e.g., Marttinen et al. 2016; Palmeroni et al. 2020; Schwartz et al. 2011; Skhirtladze et al. 2016; Zimmermann et al. 2015).

The **Identity Distress Scale** (IDS; Berman et al. 2004) was used to measure interference or severe disturbance with identity development in terms of Identity Distress (DSM‐III; American Psychiatric Association APA 1986) and Identity Problem (DSM‐IV; APA 1994). The IDS provides continuous measures for seven areas of difficulty (long term goals, career choice, friendships, sexual orientation, religion, values and beliefs, and group loyalties). Items are rated on a 5‐point Likert scale (“not at all” to “very severely”) to indicate the extent to which respondents have been recently upset, distressed, or worried over each of these identity‐related issues. Three additional items indexed the overall level of discomfort these issues caused upset or distress, how much uncertainty as a whole these issues interfered with everyday functioning, and how long, if at all, they felt upset, distressed, or worried over these issues overall. Mean scores of the IDS‐10 (ω = 0.85) were used. The IDS has been used cross‐culturally with psychometrics available (Berman and Montgomery 2014; Janowicz et al. 2024; Palmeroni et al. 2020).

The **Objective Measure of Ego Identity Status** (OMEIS‐24; Adams 1998) was used to assess the four identity statuses: foreclosure (*α* = 0.70), achievement (*α* = 0.57), moratorium (*α* = 0.65), and diffusion (*α* = 0.55) as defined by Marcia (1966). The short form has six items per scale rated on a 6‐point scale (“strongly disagree” to “strongly agree”) to indicate how each statement applies to oneself. Responses are summed to provide scores for each identity status. See Kroger and Marcia (2011) for an extensive review.

The **Identity Styles Inventory** (ISI‐3; Berzonsky 1992a) was used to measure the characteristic ways in which students process self‐relevant information about identity issues. It includes 40‐items rated on a 5‐point scale (“strongly disagree” to “strongly agree”) for three identity styles: informative (*α* = 0.65), normative (*α* = 0.59), diffuse avoidant (*α* = 0.72), and a commitment scale (*α* = 0.70) with 10‐items summed for each scale

The **Ego Identity Processes Questionnaire** (EIPQ; Balistreri et al. 1995) measured the dimensions of Exploration (*ω* = 0.62) and Commitment (*ω* = 0.67) in eight areas: occupation, religion, politics, values, family, friendships, dating, and sex roles. It consists of 32 items rated on a 6‐point scale (“strongly agree” to “strongly disagree”) summed for each scale.

The **Psychological Inventory of Ego Strengths** (PIES; Markstrom et al. 1997) assessed ego strengths or values, that is, progressive functioning in terms of Erikson's eight stages. The short form includes 32 items, four for each of the eight ego strengths (hope, will, purpose, competence, fidelity, love, care, and wisdom) rated on a 5‐point Likert scale (“does not describe me well” to “describes me very well”). Items were summed for a composite score (*ω* = 0.90). See Markstrom et al. 1997; Markstrom and Marshall 2007) for psychometrics.

The **Counseling Center Assessment of Psychological Symptoms** (CCAPS‐34; Locke et al. 2012), a screening instrument developed to address the mental health of university students, measured psychological symptoms. The CCAPS‐34 includes 34 items rated on a 5‐point scale from 0 (“not at all like me”) to 4 (“extremely like me”) to indicate the extent to which the symptom has been experienced in the past 2 weeks. The scales include depression (ω = 0.87), generalized anxiety (ω = 0.84), social anxiety (ω = 0.71), academic difficulties (ω = 0.85), eating concerns (ω = 0.86), hostility (ω = 0.84), and substance/alcohol use (ω = 0.84). Scores were computed for each scale and a Distress Index (CCAPS‐DI; ω = 0.93) provided a standardized index of scores on 20 items (Youn et al. 2015). See the CCAPS User Manual (Center for Center for Collegiate Mental Health CCMH 2015; Locke et al. 2012; McAleavey et al. 2012; Sherman et al. 2020) for psychometrics.

**Perceived global stress** was indexed by ten items from standard stress scales that indicate students' perceptions of general stress experienced over the past month (Gfellner and Córdoba 2011). Items (e.g., “In the past month how often have you thought that you could not cope with all the things you had to do?”) were rated on a 5‐point scale (“never” to “very often”) and summed (*ω* = 0.85).

**Optimism** was measured by the three positive items on the Life Orientation Test‐Revised (LOT‐R; Scheier et al. 1994). Items (e.g., “In uncertain times I usually expect the best”) are rated on a 5‐point scale (“strongly disagree” to “strongly agree”) with a high score positive (*ω* = 0.79).

**Agency** was measured by the self‐reliance scale from the four‐factor emotional autonomy model revised for use with EA (Lamborn and Groh 2009). It assesses agency to adjustment with 7‐items (e.g., “Luck decides most of the things that happen to me”) rated on a 4‐point scale (*ω* = 0.82).

The **Student Adjustment to College Questionnaire** (SACQ; Baker and Siryk 1999) measures academic (ω = 0.79), social (ω = 0.79), and personal‐emotional (PE; ω = 0.85) adaptation to university. The 27 short‐form items are rated on a 9‐point scale with end points labelled as “does not apply to me at all,” to “applies very closely to me.” The SACQ is used extensively in counseling centers and research (Credé and Niehorster 2012).

**Academic Locus of Control‐Revised** (ALOC‐R; Curtis and Trice 2013) measures the extent to which students believe that their ability and effort for success at university are under their own control (internal) or the control of external sources. The 21‐item scale is answered in a true/false format (e.g., “For some courses, it is not important to go to class”). High scores indicate an external orientation (ω = 0.72).

The **Academic Entitlement Questionnaire** (AEQ; Kopp et al. 2011) measures the extent to which students feel that they are privileged in academic settings regardless of effort. It is a key variable in academic success. Eight items (e.g., “It is the professor's responsibility to make it easy for me to succeed.”) are rated on a 7‐point scale (“strongly disagree” to “strongly agree”). (ω = 0.84)

Procedure. A repeated cross sectional (RCS) study provided data collected in 2017/18. Instructors invited students to volunteer to complete a survey available on class websites. Students were awarded a bonus point as a gratuity for participation. The project received ethical approval from the Brandon University Research Ethics Committee (Certificate #200325).

Data Analysis. The exclusionary criterion eliminated participants with more than two missing items from the DIDS and the IDS to meet the MPlus analysis requirements. Latent profile analyses were then conducted in MPlus to generate a series of identity status groups. These groups were then compared in terms of theoretical meaningfulness, parsimony, and explanatory power. When the appropriate groups were identified, profile ANOVAs were run for all variables in the study.

## 8 Results

**Figure 1:**
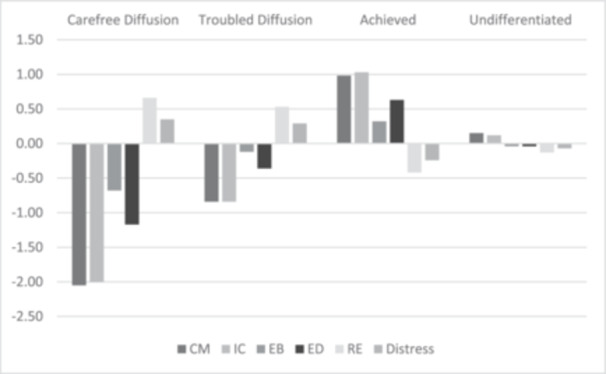
Latent profiles of students in Canada. *Note:* CM = Commitment Making, IC = Identification with Commitment, EB = Exploration in Breadth, ED = Exploration in Depth, RE = Ruminative Exploration, Distress = Identity Distress (IPS).

The five profile solutions generated for the DIDS and IDS variables are shown in Supporting Table 2. Following the latent analysis guidelines (Spurk et al. 2020) the four‐profile solution was retained. The identified statuses were: Carefree Diffusion (6.9%), Troubled Diffusion (24.8%), Achievement (28.2%), and Undifferentiated (40.1%). These profiles align with statuses reported in other studies (Luyckx et. 2008a, 2008b; Marttinen et al. 2016; Schwartz et al. 2011; Skhirtladze et al. 2016). Consistent with Schwartz et al. (2011) for US students, undifferentiated was the largest group. However, there were neither foreclosure nor moratorium in the Canadian student profiles. Figure 1 illustrates the alignment of the identity dimensions relative to mean scores for the profiles.

Table 1 shows the identity profile ANOVA results for the DIDS and IDS scores. As expected, the groups differed significantly on the commitment and exploration scales. For the maladaptive processes (RE and IDS) the carefree and troubled diffusion groups scored significantly above the undifferentiated group whose scores exceeded those of the achieved students.

**Table 1:** Summary of the Predictors of the Person‐Centered Profiles for Canadian Students.

Variable	Sample mean	Carefree diffusion	Troubled diffusion	Achieved	Undifferentiated	*F*‐value	Eta^2^
		6.9% (59)	24.8% (233)	28.2% (261)	40.1% (361)		
**DIDS** ^**1**^							
CM	3.71 (0.93)	1.66 (0.44)^d^	2.87 (0.42)^c^	4.69 (0.31)^a^	3.86 (0.33)^b^	1750.98***	0.85
IC	3.74 (0.92)	1.74 (0.44)^d^	2.90 (0.38)^c^	4.77 (0.26)^a^	3.86 (0.31)^b^	2099.1***	0.88
EB	3.70 (0.70)	3.02 (1.1)^c^	3.58 (0.66)^b^	4.02 (0.67)^a^	3.66 (0.53)^b^	44.04***	0.13
ED	3.53 (0.73)	2.36 (0.75)^d^	3.17 (0.56)^c^	4.16 (0.55)^a^	3.49 (0.49)^b^	236.2***	0.44
RE	2.92 (0.88)	3.58 (0.96)^a^	3.45 (0.69)^a^	2.50 (0.89)^c^	2.79 (0.75)^b^	74.6***	0.20
**Identity distress**	2.28 (0.74)	2.63 (0.8)^a^	2.57 (0.7)^a^	2.04 (0.7)^c^	2.21 (0.7)^b^	27.18***	0.09
**Validation**							
Diffusion^2^	3.49 (0.96)	4.08 (1.9)^a^	3.82 (0.92)^a^	3.23 (0.98)^b^	3.36 (0.87)^b^	17.31***	0.08
Foreclosure^2^	2.53 (1.0)	2.61 (1.2)^a^	2.59 (0.94)^a^	2.33 (1.04)^b^	2.62 (0.90)^a^	3.15*	0.01
Moratorium^2^	2.89 (0.96)	3.31 (0.94)^a^	3.37 (0.94)^a^	2.47 (0.88)^c^	2.80 (0.85)^b^	29.99***	0.13
Achieved^2^	3.80 (0.84)	3.26 (0.93)^c^	3.51 (0.73)^c^	4.16 (0.91)^a^	3.82 (0.73)^b^	22.68***	0.11
Informative^3^	3.33 (0.48)	3.24 (0.5)^bc^	3.21 (0.49)^c^	3.47 (0.47)^a^	3.32 (0.46)^b^	13.37***	0.04
Normative^3^	3.15 (0.54)	2.89 (0.54)^c^	2.99 (0.54)^c^	3.33 (0.57)^a^	3.17 (0.50)^b^	22.91***	0.07
Diffuse^3^	2.55 (0.58)	2.70 (0.49)^a^	2.81 (0.49)^a^	2.32 (0.62)^c^	2.51 (0.53)^b^	34.66***	0.10
Commitment^3^	3.45 (0.66)	2.86 (0.74)^c^	2.97 (0.52)^c^	3.89 (0.54)^a^	3.53 (0.51)^b^	143.45***	0.32
Ego strengths^4^	3.62 (0.54)	3.08 (0.57)^d^	3.30 (0.43)^c^	4.0 (0.52)^a^	3.65 (0.43)^b^	99.09***	0.27
Exploration^5^	56.2(9.3)	57.8 (8.8)	56.3 (8.1)	56.6 (8.1)	55.7 (8.6)	1.03	0.00
Commitment^5^	60.3 (8.9)	54.3 (10.8)^c^	56.0 (7.1)^c^	64.6 (8.7)^a^	61.0 (8.1)^b^	52.5***	0.15
**Qutcomes**							
Academic^6^	6.52 (1.2)	5.70 (1.25)^c^	5.85 (1.10)^c^	7.15 (0.98)^a^	6.64 (1.01)^b^	78.04***	0.21
Social^6^	5.43 (1.3)	4.98 (1.45)^c^	5.11 (1.28)^c^	5.79 (1.33)^a^	5.45 (1.32)^b^	13.88***	0.04
P‐Emotional^6^	4.9 (1.7)	4.41 (1.59)^a^	4.40 (1.61)^a^	5.16 (1.72)^a^	5.10 (1.72)^a^	12.25***	0.04
Global stress	2.85 (0.71)	3.11 (0.81)^a^	3.05 (0.64)^a^	2.70 (0.73)^b^	2.80 (0.68)^b^	14.05***	0.05
CCAPS^**7**^	1.04 (0.70)	1.42 (0.82)^a^	1.09 (0.71)^a^	0.65 (0.65)^b^	0.77 (0.68)^b^	24.33***	0.08
A‐LOC^**8**^	7.40 (3.5)	10.37 (3.5)^a^	9.23 (3.30)^b^	6.69 (3.2)^d^	7.00 (3.1)^d^	68.69***	0.19
Agency	3.21 (0.51)	3.17 (0.44)^a,b,c^	3.00 (0.62)^c^	3.40 (0.43)^a^	3.21 (0.43)^b^	10.19***	0.09
Optimism	3.33 (0.9)	2.21 (0.93)^c^	3.09 (0.82)^b^	3.88 (0.88)^a^	3.28 (0.76)^b^	52.8***	0.06
Entitlement	2.73 (1.2)	2.71 (1.2)^a,b^	2.94 (1.23)^a^	2.57 (1.24)^b^	2.70 (1.05)^a,b^	4.15**	0.01

*Note:* ^1^ = Dimensions of Identity Development Scales; ^2^ = Extended Objective Measure of Ego Identity Status Scales; ^3^= Identity Styles scale & commitment; ^4^ = Psychological Inventory of Ego Strengths; ^5^ = Ego Identity Process scales; ^6^ = Student Adjustment to College scales; ^7^ = Counseling Center Assessment of Psychological Symptoms, ^8^ = Academic Locus of Control: high score = externality; ***p < 0.001; **p < 0.01; *p < 0.05; ^a,b,c,d^ different superscripts indicate significant mean differences.

The identity profiles were then examined in relation to established measures of identity. A series of ANOVAs by profile were run for the OMEIS‐24 (Adams 1998), Ego Identity Processes (Balistreri et al. 1995), Identity Styles and Commitment (Berzonsky 1992b), and the Psychological Inventory of Ego Strengths (Markstrom et al. 1997). Psychosocial maturity on these scales was expected to align with advanced development on the identity profiles. The results are given in Table 1. As predicted, ego strength scores declined significantly across the four profiles with the highest score for achievement, followed by undifferentiated, troubled diffusion, and carefree diffusion, respectively. For the other identity scales, the greatest maturity scores were for achievement followed by undifferentiated then the troubled and carefree diffusion groups (which did not differ). This included positive scores for: OMEIS foreclosure and achievement; informative and normative identity styles and commitment; and IPQ‐commitment. Alternatively, the troubled and carefree diffusion groups scored highest on the least adaptive identity measures (OMEIS diffusion and moratorium; diffuse identity style).

Age and sex differences were examined across the profiles for comparison with other studies. Age differences were not found, *F* (3, 912) = 0.965, *p* < 0.41. Although the Chi square for sex did not attain significance, *χ* ^2^ (3 df) = 6.99, *p* < 0.07, consistent with Schwartz et al. (2011) more women (30.5%) than men (21%) were in the achieved profile.

The next set of analyses examined the identity profiles in relation to functional well‐being, including (1) adaptive functioning at university (academic, social, personal‐emotional, academic locus of control, academic entitlement), and (2) mental health (optimism, agency/reliance, general stress, and psychological symptoms/CCAPS).

Table 1 shows the results of the profile ANOVAs for the adaptive functioning at university measures. As expected, achieved students indicated elevated academic and social adjustment at university, followed by the undifferentiated, and these students scored significantly higher than the troubled diffusion and carefree diffusion (who did not differ). Alternatively, the achieved and undifferentiated groups did not differ on personal‐emotional adjustment at university scores, and their scores significantly exceeded those of the two diffusion groups. This is consistent with psychological symptoms on the CCAPS. As expected, students' academic locus of control scores differed significantly in relation to maturity of the identity profiles with externality highest for the carefree diffused, followed by the troubled diffused, and the undifferentiated and achieved groups (did not differ). Academic entitlement scores were greatest for troubled diffusion students and differed only from achieved students.

As seen in Table 1 there was directional support for the mental health variables across the profiles. However, only optimism scores differed significantly between the groups with the highest scores for achieved students followed the undifferentiated, troubled diffusion, and carefree diffusion groups, respectively. Comparably achieved students' agency scores exceeded those of undifferentiated students, and these students scored significantly higher than the troubled diffusion group, but carefree diffusion students' scores did not differ from either of these adjacent groups. Students in the diffused groups reported the greatest global stress scores in comparison with the undifferentiated and achieved groups. As noted above similar differences were found with elevated scores for psychological symptoms (CCAPS‐DI) and scales for depression, general anxiety, social anxiety, eating concerns, and hostility among students in the two diffusion groups (Supporting Table 3). As with high academic and social adjustment on the SACQ, achieved students scored lowest on CCAPS‐academic difficulties, followed by undifferentiated students who scored significantly below both diffusion groups.

## 9 Discussion

This study provided a latent profile analysis of identity processes with identity distress, the affective component of identity among Canadian university students. Identity distress was an additional maladjustment component that reflected uncertainty, upset, worry, and discomfort with relevant identity concerns (Berman 2020). The profiles showed some alignment with those reported in the United States (Johnson 2012; Schwartz et al. 2011) and other countries (Hatano and Sugimura 2017; Luyckx et al. 2008a, 2008b; Luyckx, Klimstra, Schwartz, et al. 2013; Mannerström et al. 2017; Raemen et al. 2022; Vankerchoven et al. 2023). Consistent with the identity framework, the achieved profile scored highest on the adaptive identity processes and lowest on RE and identity distress.

The carefree diffused scored significantly below troubled diffusion on the adaptive dimensions indicating no consideration of identity work (Schwartz et al. 2011). However, significantly higher scores for the exploration and commitment processes among troubled diffusion students as well as consistency with the undifferentiated group on EB may be an early precursor of identity construction (Schwartz et al. 2011). Nevertheless, the similar low RE and IDS scores for the diffused groups are unclear and may indicate differences in relation to potential identity unfolding. These negative identity components may function constructively for troubled diffusion students as a stimulus for exploration. Alternatively, RE and identity distress are expected to interfere maladaptively as an impediment for carefree diffused individuals with no interest in identity concerns, yet these students may be confronted with other stressful aspects in the university context that are reflected in identity distress and RE. As discussed later, diffused students are at greater risk for serious psychological problems that impede identity development (Gfellner and Cordoba 2020a; Klimstra and Denissen 2017). Follow‐up of students in these profiles is necessary to determine how identity work may be resolved uniquely or whether carefree diffusion is a subset of troubled diffusion among these Canadian students.

The undifferentiated students scored close to the sample means on each of the dimensions. It is consistent with what Adams (1998) labelled “low profile moratorium” (Schwartz et al. 2011). This category was used to classify cases that did not fit distinctively into any of the identity statuses. Although these individuals show some proclivity for identity development, they reflect a reticent approach to proactive identity involvement (Adams et al. 2006).

Luyckx et al. (2008a) found undifferentiated but no moratorium clusters in samples of university freshmen and 12th grade Belgian students. This is consistent with the lack of a moratorium profile in the current sample with undifferentiated, the largest group. Similarly, undifferentiated clusters accounted for the greatest proportion of university students in the US (Schwartz et al. 2011; Johnson 2012), Belgium (Luyckx et al. 2008a) and a Georgian sample (Skhirtladze et al. 2016).

In a 16‐month follow‐up with college students Johnson (2012) found that the undifferentiated and moratorium clusters were least stable (in comparison with Confident Committers/Foreclosed and Questioning Achievers/Achieved with higher than expected RE). Students in these profiles were likely to transition to one of the committed profiles. Similarly, the sizable undifferentiated profile in the current study may precede a transitional phase in the progression of identity construction.

Hatano and Sugimura (2017) reported that Japanese adolescents transitioned from troubled diffused to moratorium and from moratorium to achieved in a 4‐year study during the secondary school years with neither foreclosed nor undifferentiated profiles. These findings suggest preliminary phases in the onset of exploration during adolescence and early EA. In the current study, apart from the achieved, there was little evidence of commitment or exploration, and a foreclosed profile was not found. Similar results with adolescents (Hatano and Sugimura 2017; Vankerckhoven et al. 2023) and university women in Belgium (Luyckx et al. 2008a) were interpreted as due to sample and context characteristics. Alternatively, undifferentiated profiles were absent in two longitudinal studies with women (Luyckx et al. 2008a), youth and adolescents (Raemen et al. 2022; Vankerchoven et al. 2023), college and working young adults in Belgium (Luyckx, Klimstra, Schwartz, et al. 2013), and Finnish EAs (Mannerström et al. 2017). Such variability in these profiles has been associated broadly with differences in samples such as individual/demographic, social/contextual, and country/cultural factors. According to these researchers it is not uncommon that not all identity profiles are identified in every specific study. Indeed, there was no Drifter (diffused) profile in the Luyckx et al. (2008a) longitudinal study of college women. In many studies samples tend to be relatively homogeneous in background and contextual characteristics. Alternatively, North American samples have greater diversity in terms of race and ethnicity, SES, program of studies, region, living situations, and cultural ideology. However, most research has emphasized individualism (*vs.* collectivism) without consideration of other cultural dimensions (Hofstede 2015). A cultural interpretation of Finnish EA profiles (Marttinen et al. 2016) provides a comparison with current findings.^1^

In terms of culture and context, the current sample is in a nonmetropolitan city of a less densely populated province with smaller communities and rural agriculture. It may involve somewhat of the rural‐urban gap in higher education which reflects difficulties experienced by rural students (McCauley 2025; Wood 2023). In terms of regional differences (Statistics Canada 2022), the earlier age of leaving one's parents' home and community portends less supervision, structure, guidelines, and unfamiliarity of the environment thereby increasing distress and obstacles to identity work (Arnett et al. 2014). This regional context may be reflected in students delayed identity development seen in the lack of moratorium and foreclosed profiles. Such divergence from normative identity paths has been reported in Japan (Hatano and Sugimura 2017) and Georgia (Skhirtladze et al. 2016) and interpreted in terms of social change, regional, and cultural differences. These findings indicate alternative routes in identity development and underscore the need to investigate subtle social and cultural factors in different regions of Canada, the US, and other countries (Galliher et al. 2017).

Clear differentiation across the profiles for ego strengths reflected psychosocial maturity in terms of Erikson's stages (Markstrom et al. 1997). For the other identity measures the alignment was supported for the achieved and undifferentiated but not the diffused profiles. The lack of differentiation of EIP‐exploration scores across the profiles may reveal the confound between reflective and dysfunctional components of exploration as indicated in the dual‐cycle model (Schwartz et al. 2011). As indicated in the configuration of profiles, Canadian students are young EAs in a formative phase of identity development. The university context provides an important stimulus for exploration, yet the lack of structure, direction, and increased freedom creates considerable uncertainty that interferes with identity development for many (Arnett et al. 2014). These students are in a liberal arts and sciences program, are not required to select a major until the second year or later, and many have relocated from a rural setting. Apart from the achieved, most students are on the threshold of exploration. From the cultural perspective, the country's indulgent work‐leisure balance with its emphasis on fun in the context of individualism (Hofstede 2015) along with the newfound freedom and lack of structure in the university environment may postpone an immediate focus on identity development.

Consistent with other studies, the mental health measures were associated with mature identity development (Schwartz et al. 2011, 2013). Optimism was highest among achieved students; the lowest score for the carefree diffused may indicate the lack of reflective exploration (Schwartz et al. 2011) and focus on immediate “here and now” gratification. Higher scores for the troubled diffusion and undifferentiated students may be a precursor to forthcoming identity work. This is consistent with developmental individuation that includes agenic capacities (i.e., ego strengths, optimism, locus of control, agency/reliance) that are positively related to exploration and flexible commitment (Schwartz et al. 2005).

There was directional support for psychological symptoms of achieved and undifferentiated students with scores in the normal range (Youn et al. 2015). This may provide a backdrop for identity development among the undifferentiated. Conversely, the carefree diffusion score of 1.43 exceeds the screening cutoff (1.0) for moderate clinical severity with the troubled diffused score somewhat lower (1.26). As shown in Supplementary Table 3, this differentiation was seen for the CCAPS subscales. In comparison Schwartz et al. (2011) found no differences in general and social anxiety between the two diffused profiles in their US sample. Alternatively, Belgian troubled diffusion students had higher depression scores than their carefree diffused cohort (Luyckx et al. 2005). This was considered a cultural artifact (Schwartz et al. 2013). Increased alcohol and drug problems among the carefree diffused aligned with Schwartz et al. (2011).

As with psychological symptoms, undifferentiated students' lower perceived global stress may be part of a constellation of assets that herald identity advancement. Nevertheless, given the increased incidence of psychological difficulties among university students (Balon et al. 2015) these findings underscore the need for closer attention to mental health to unravel some of the complexities in EA's identity development. Similarly, Raemen et al. 2022) emphasized the importance of identity development in the prevention and treatment of psychopathology.

The university environment provides an important context with encouragement and opportunities to enhance identity development (Arnett et al. 2014; Cote 2006). As predicted, the results supported advanced psychosocial maturity, optimism, academic locus of control, academic and social functioning among achieved students. However, as with psychological symptoms, consistency of personal‐emotional adjustment at university of the undifferentiated relative to achieved students further anticipates a readiness for identity construction. Similarly, the lack of significant differences in psychological symptoms between the diffused groups requires careful consideration as it reflects consistency in the maladaptive components rather than adaptive identity processes. This requires longitudinal study. Nevertheless, focused investigation of associations between identity development and mental health offers both theoretical and practical clarification for the similarity of psychological symptoms in the differentiated and achieved profiles.

Several limitations of this study require consideration. First, the use of a repeated cross‐sectional study enables comparison of successive cohorts, but it does not allow for the examination of prospective change over time. Further research would benefit from longitudinal follow‐up of students to elucidate transitions in identity development and how they are related to relevant functional outcomes. Second, this study relied on self‐report measures. Qualitative measures would provide more nuanced information on relevant components of identity functioning and related aspects of mental health and functional abilities. In addition, external sources of information on psychological problems and academic records would provide objective information to supplement subjective reporting of respondents. As noted previously the higher proportion of women to men reflects the sex distribution in psychology and general introductory classes. It is a consistent drawback of research with college samples (Salkind 2010).

## Supporting information


**Supporting Table 1:** Demographic characteristics of the Canadian students.

**Supporting Table 2:** Latent Profile Analysis Statistics and Fit Indices for Canada (n = 914).

**Supporting Table 3:** ANOVAs for the CCAPS scales by the Person‐centered Profiles.

## Endnotes

1 Finland resembles Canada on Hofstede (2015) indulgent‐restraint cultural dimension that emphasizes a salient work‐leisure lifestyle balance. In a sample of diverse Finnish EAs, Marttinen et al. (2016) reported several profiles using the abbreviated DIDS that were identified differently but appear similar to several profiles in the current study. The largest profile (43.5%), Moderate Achievement with DIDS scores near the sample means (no extreme scores on any scales) resembled an Undifferentiated configuration. This profile was similar to the Achieved profile in associations with outcome measures. Neither Foreclosed nor Moratorium profiles were found. Two diffusion profiles were labelled: Moderate Diffusion and Diffuse (Troubled) Diffusion. These profiles were less extreme than the diffused profiles in the current study and indicated consistent differences over a 2‐year period in normative and atypical goals and concerns. However, apart from the indulgent cultural dimension, other differences are important in the interpretation of profiles. For example, the Finnish study used a short form DIDS with a modified ED that is, ED‐R (reconsidered), the EA sample was diversified, and the Finnish postsecondary educational system is stringent and competitive.
