# Supplementary material for: Extending the Dark Side of Identity Processes With Identity Distress
Source: J Adolesc. 2025 Aug 11;97(8):2226–35. doi: 10.1002/jad.70034 (PMC12682240; doi:10.1002/jad.70034)
Supplement: Supplementary file 1 — Supporting Table 1: Demographic characteristics of the Canadian students. [file JAD-97-2226-s002.docx]

Supplementary Table 1. Demographic characteristics of the Canadian students

_______________________________________________________________________

Age (years) 19.43 (2.1)

Sex

Male 20.3%

Female 79.7%

Ethnicity/Culture

Caucasian 79.4%

Indigenous 7.5%

Asian 5.9%

Black 3.4%

International 3.2%

Other 0.6%

Marital Status

Single 94.3%

Married/Common Law 5.3%

Divorced 0.4%

Year in University

First 63.5%

Second 21.6%

Third 9.3%

Fourth 4.2%

Fifth or higher 1.1%

Home Community

Same community 34.8%

Same province 52.0%

Another province 9.6%

Another country 3.6%

Living situation

With parents 36.8%

Spouse/common law partner 6.1%

With roommates (house, apt.) 32.9%

Residence/dorm 13.0%

Alone 8.4%

Home with children 0.7%

Other 2.1%

Parental education attained

Maternal median level Some university or college

Paternal median level Some university or college
