# Supplementary material for: Extending the Dark Side of Identity Processes With Identity Distress
Source: J Adolesc. 2025 Aug 11;97(8):2226–35. doi: 10.1002/jad.70034 (PMC12682240; doi:10.1002/jad.70034)
Supplement: Supplementary file 2 — Supporting Table 2: Latent Profile Analysis Statistics and Fit Indices for Canada (n = 914). [file JAD-97-2226-s001.docx]

*Table 1. Latent Profile Analysis Statistics and Fit Indices for Canada (n = 914)*

|  |  |  |  | Classification Probabilities | | | | | Profile Size (%) | | | | |
| --- | --- | --- | --- | --- | --- | --- | --- | --- | --- | --- | --- | --- | --- |
| Profiles | AIC | BIC | Entropy | 1 | 2 | 3 | 4 | 5 | 1 | 2 | 3 | 4 | 5 |
| 1 | 13065.82 | 13123,46 |  | 1 |  |  |  |  | 914  (100) |  |  |  |  |
| 2 | 11767.28 | 11858.82 | 0.83 | 0.95 | 0.97 |  |  |  | 319  (34.9) | 595  (65.1) |  |  |  |
| 3 | 11248.82 | 11374.09 | 0.84 | 0.91 | 0.94 | 0.92 |  |  | 295  (32.2) | 454  (49.7) | 165  (18.1) |  |  |
| **4** | **10739.87** | **10898.86** | **0.90** | **0.90** | **0.95** | **0.96** | **0.93** |  | **59**  **(6.5)** | **233**  **(25.5)** | **261**  **(28.6)** | **361**  **(39.5)** |  |
| 5 | 10552.68 | 10745.40 | 0.90 | 0.94 | 0.92 | 0.88 | 0.92 | 0.95 | 360  (39.4) | 40  (4.4) | 39  (4.3) | 221  (24.18) | 254  (27.8) |
