# Supplementary material for: Extending the Dark Side of Identity Processes With Identity Distress
Source: J Adolesc. 2025 Aug 11;97(8):2226–35. doi: 10.1002/jad.70034 (PMC12682240; doi:10.1002/jad.70034)
Supplement: Supplementary file 3 — Supporting Table 3: ANOVAs for the CCAPS scales by the Person‐centered Profiles. [file JAD-97-2226-s003.docx]

Supplementary Table 3. ANOVAs for the CCAPS scales by the Person-centered Profiles

| Variable | Sample Mean | Profile 1 Carefree **Diffusion** | Profile 2 Troubled **Diffusion** | Profile 3 **Questioning Achievement** | Profile 4 **Undifferentiated** | F-value | Eta^2^ |
| --- | --- | --- | --- | --- | --- | --- | --- |
|  |  | 6.9% (59) | 24.8% (233) | 28.2% (261) | 40.1% (361) |  |  |
| **CCAPS Scales** |  |  |  |  |  |  |  |
| Depression | 0.84(.72) | 1.22(.82)a | 1.09(.71)a | 0.65(.65)b | 0.77(.68)b | 24.33*** | .075 |
| General Anxiety | 0.98(.85) | 1.12(.84)a | 1.19(.90)a | 0.85(.80)b | 0.92(.82)b | 7.84*** | .025 |
| Social Anxiety | 1.47(.76) | 1.86(.75)a | 1.63(.74)a | 1.31(.77)b | 1.41(.74)b | 12.62*** | .043 |
| Academic Difficulties | 1.31(.74) | 1.66(1.2)a | 1.53(.76)a | 1.10(.72)c | 1.26(.69)b | 20.01*** | .062 |
| Eating Concerns | 1.07(.99) | 1.45(1.2)a | 1.30(1.1)a | 0.92(.95)b | 0.97(.87)b | 10.66*** | .034 |
| Hostility | 0.57(.66) | 0.72(.72)a | 0.73(.72)a | 0.42(.49)b | 0.55(.63)b | 10.31*** | .033 |
| Alcohol & Drugs | 0.55(.69) | 0.55(.68)ab | 0.72(.84)a | 0.47(.58)b | 0.49(.65)b | 6.98*** | .023 |

Notes. *** p < .001; ^abc^ different superscripts indicate differences between the means
